# Esophageal atresia in newborns: a wide spectrum from the isolated forms to a full VACTERL phenotype?

**DOI:** 10.1186/1824-7288-39-45

**Published:** 2013-07-10

**Authors:** Simona La Placa, Mario Giuffrè, Antonella Gangemi, Stefania Di Noto, Federico Matina, Federica Nociforo, Vincenzo Antona, Maria Rita Di Pace, Maria Piccione, Giovanni Corsello

**Affiliations:** 1 Department of Sciences for Health Promotion and Mother and Child Care, Università di Palermo, Via Alfonso Giordano 3, Palermo, 90127, Italy

**Keywords:** Esophageal atresia, Tracheo-esophageal fistula, VATER, VACTERL, Association, Congenital malformations, Anomalies

## Abstract

**Background:**

VATER association was first described in 1972 by Quan and Smith as an acronym which identifies a non-random co-occurrence of Vertebral anomalies, Anal atresia, Tracheoesophageal fistula and/or Esophageal atresia, Radial dysplasia. It is even possible to find out Cardiovascular, Renal and Limb anomalies and the acronym VACTERL was adopted, also, embodying Vascular, as single umbilical artery, and external genitalia anomalies.

**Methods:**

Data on patients with esophageal atresia (EA) with or without tracheoesophageal fistula (TEF) admitted in the Neonatal Intensive Care Unit (NICU) between January 2003 and January 2013 were evaluated for the contingent occurrence of typical VACTERL anomalies (VACTERL-type) and non tipical VACTERL anomalies (non-VACTERL-type). The inclusion criterion was the presence of EA with or without TEF plus two or more of the following additional malformations: vertebral defects, anal atresia, cardiovascular defects, renal anomalies and lower limb deformities, like radial dysplasia.

**Results:**

Among 52 patients with EA/TEF, 20 (38,4%) had isolated EA and 7 (21,8%) had a recognized etiology such a syndrome and therefore were excluded. Among 32 infants with EA and associated malformations, 15 (46,8%) had VACTERL association. The most common anomalies were congenital heart defects (73,3%), followed by vertebral anomalies (66,6%). Many patients also had additional non-VACTERL-type defects. Single umbilical artery was the most common one followed by nervous system abnormalities and anomalies of toes. Between the groups of infants with VACTERL type and non-VACTERL-type anomalies, there are several overlapping data regarding both the tipically described spectrum and the most frequently reported non-VACTERL-type malformations. Thus, it is possible to differentiate infants with a full phenotype (VACTERL full phenotype) and patients that do not meet all the criteria mentioned above, but with some homologies with the first group (VACTERL partial phenotype).

**Conclusion:**

The high frequency of non-VACTERL-type anomalies encountered in *full* and *partial* phenotype patients would suggest the need for an extension of the clinical criteria for the diagnosis of VACTERL association and also for pre- and post-operative management and follow-up in the short and long term.

## Background

VATER association is a specific pattern of multiple congenital malformations [1] and was first described in 1972 by Quan and Smith as a non-random co-occurrence of Vertebral anomalies, Anal atresia, Tracheoesophageal fistula and/or Esophageal atresia, Radial dysplasia. All single cases, did not show history of teratogenic etiologies or chromosomal aberrations [2-4]. The original acronym VATER has been widened into VACTERL, including Cardiovascular, Renal and Limb anomalies associated with the former ones [2,5-7]. Later, other authors suggested that diagnostic criteria should include Vascular anomalies (such as single umbelical artery) as part of the V in the acronym and other malformations (such as external genitalia and laryngotracheal anomalies) [2,8-12]. Furthermore, it was stated that VACTERL association would incorporate just the preaxial limb anomalies [13]. The inclusion of cardiac defects is still debated, however, as is the number of defects needed for the diagnosis of VACTERL association [13-19]. In 2001, other authors divided patients in an “upper” and a “lower” group, on the basis of the localization of their own anomalies as like as upper ones (cardiac, esophageal or limb deformities) and lower ones (caudal dysgenesis, renal, anorectal and lower vertebral anomalies) [16]. In a later study, patients with similar phenotypes were grouped in five peculiar clusters [20]. Since there is a wide margin of interpretation in diagnostic criteria, a thorough examination is mandatory in all patients with the suspicious of VACTERL association. In some studies the frequency was estimated between 1/10,000 and 1/40,000 infants [2]. Some studies have suggested that the condition is more common in males [21,22]. No specific distribution or geographic predominance in ethnic groups has been observed.

Because of the high clinical variability and sporadic occurrence, the etiology of VACTERL association is still unclear and a precise etiological cause has been identified just in a small fraction of patients so far (in example patients with hydrocephalus, VACTERL-H) [2].

In this study, data on 52 newborns with esophageal atresia (EA) with or without tracheoesophageal fistula (TEF) are shown in order to evaluate the contingent occurrence of typical VACTERL anomalies (VACTERL-type) and non typical VACTERL anomalies (non-VACTERL-type) such as single umbilical artery (SUA), duodenal atresia or stenosis, cleft lip and palate, genital anomalies, urinary tract abnormalities, hypothyroidism, anomalies of toes, lower limb anomalies and anomalies of respiratory, intestinal, vascular and nervous system.

## Methods

We aim to evaluate data collected from our Neonatal Intensive Care Unit (NICU) in Palermo University, in which are referred most of newborns coming from all Western Sicily with congenital malformations requiring a surgical treatment. We enrolled in this retrospective analysis all newborns admitted between January 2003 and January 2013. We evaluated many variables such as sex, gestational age, body weight, length and cranial circumference, APGAR score, type of EA (according to Vogt-Roberts classification), VACTERL and non-VACTERL associated anomalies.

According to de Jong et al. [21], the inclusion criteria for the diagnosis of VATER/VACTERL association were the presence of EA with or without TEF and at least two of the following defects: vertebral anomalies (V), anal atresia (A), cardiovascular malformations (C), renal anomalies (R) and limb deformities (L). The diagnostic process included an ultrasound screening (cardiac, abdominal and cerebral ultrasonography), together with X-rays of spine and limbs. Karyotyping was performed in all children with multiple malformations, in order to identify patients with chromosomal abnormalities. In addition, in some patients, further molecular and cytogenetic analysis, like array-Comparative Genomic Hybridization (a-CGH) and FISH (Fluorescence In Situ Hybridization), were performed in order to assess potential microdeletions.

## Results

**Figure 1:**
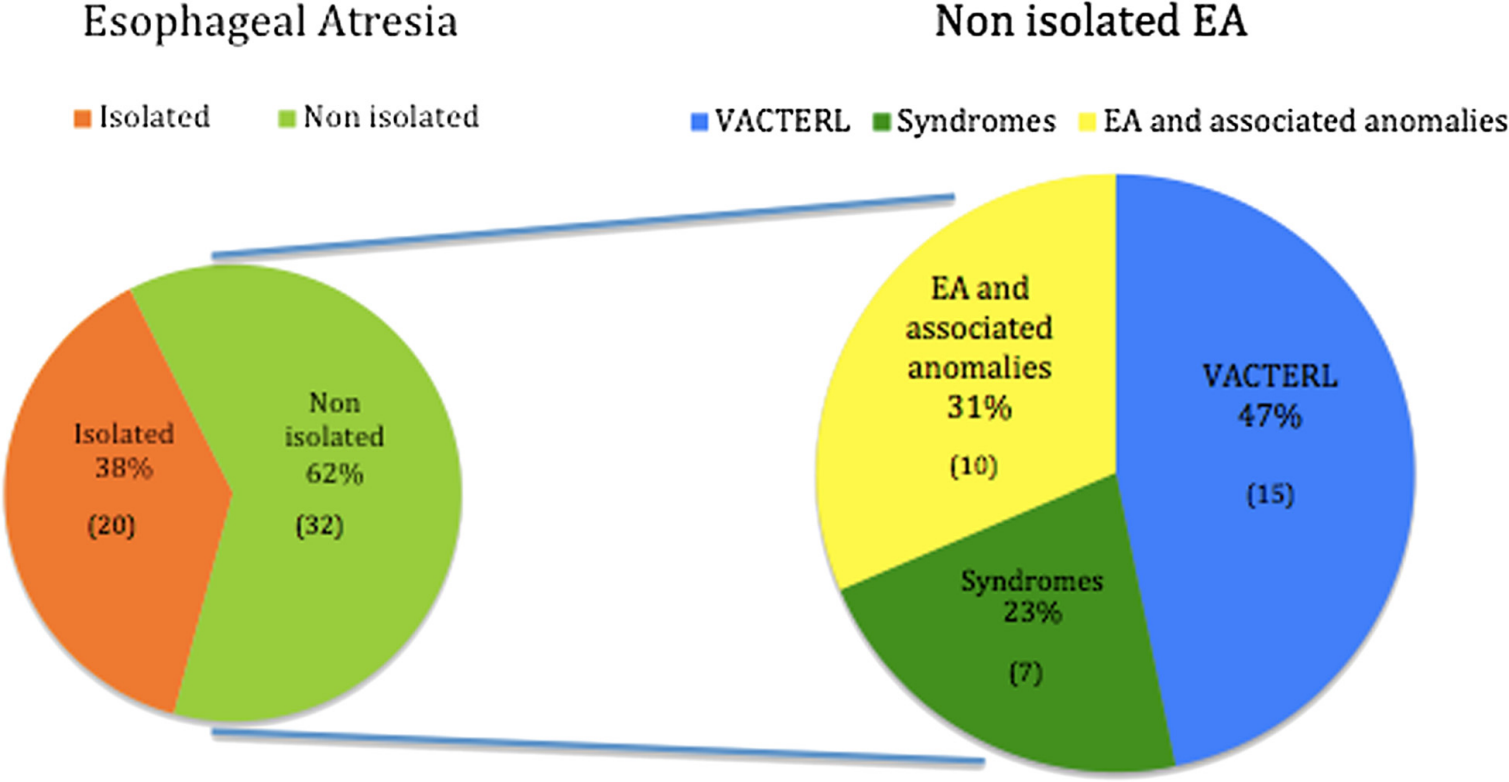
**Patients with EA/TEF admitted in this study.** Clustering of non-isolated-EA in syndromes, VACTERL association and EA with multiple malformations.

Fifty-two patients have been enrolled in the study, 20 of wich (38,4%) had isolated EA and were therefore excluded from the subsequent analysis. Among 32 infants (61,5%) with EA and associated malformations, 22 patients showed a recognizable pattern of multiple congenital anomalies (associations, syndromes or sequences), in particular 7 (21,8%) with malformation syndromes or sequences (4 with Down syndrome, 2 with Nager syndrome and 1 with CHARGE syndrome), 15 with VACTERL associations (46,8%) and 10 with EA and other multiple malformations (31%; Figure 1). Feingold syndrome and other syndromes have been considered and excluded after accurate evaluation by an experienced clinical genetitian, who has personally addresses the clinical diagnosis and the eventual need of subsequent genetical investigations.

The 15 infants with VACTERL association, 8 males and 7 females, showed EA associated with at least two other malformations included in the classic phenotypical spectrum (Additional file 1). Only in 1 patient (6,6%) the full spectrum with six anomalies was observed, whereas 4 patients (26,6%) showed four defects and 10 patients (66,6%) three. Fourteen infants (93,3%) were preterm, namely 6 were born at < 34 weeks of gestation and 8 were late-preterm, born between the 34 and 37 weeks of gestation. One neonate was born full-term at 39,3 weeks of gestation. The birth weight ranged between 1490 and 2870 g, body length between 40 and 51 cm and cranial circumference between 28.5 and 33.5 cm. Eight infants were small for gestational age and seven were adequate for gestational age. The APGAR score was normal (between 8 and 10) for all patients, except 3 newborns who required intubation and/or intravenous adrenaline (Apgar score < 7). For all patients no parental consanguineity or familial recurrence for genetics disorders were found. All 15 infants with VACTERL association had a normal karyotype; three newborns also showed normal array-CGH analysis.

Table 1 displays the prevalence of the most frequent defects observed in our patients: vertebral anomalies were found in 10 patients (66,6%), 6 of which presented also rib abnormalities. Seven patients (46,6%) had anorectal malformations and specifically five anal atresia/stenosis and two anal anteriorization. Regarding congenital heart defects (11 patients, 73,3%), we observed 5 ventricular septal defects (VSD), 3 tetralogies of Fallot, 3 patent ductus arteriosus, 4 aortic arch defects and 2 atrial septal defects (ASD). Three patients (20%) showed renal malformations such as agenesis, dysgenesis and ectopia. Six patients (40%) had abnormalities of the upper limbs and more precisely 3 of them showed defects in forearm (radius/ulna) bones while the other 3 patients showed isolated thumb anomalies.

**Table 1:** VACTERL-type defects observed in 15 newborns with esophageal atresia

**Defect**	**N. of patients**	**%**
VERTEBRAL	10	**66,6**
- Vertebral anomalies	8	53,3
- Rib anomalies	6	40
ANAL	7	**46,6**
- Anal atresia/stenosis	5	33,3
- Abnormal placement of anus	2	13,3
CARDIOVASCULAR	11	**73,3**
- Atrial septum defect	2	13,3
- Ventricular septum defect	5	33,3
- Tetralogy of Fallot	3	20
- Aortic arch defects	4	26,6
- Patent ductus arteriosus	3	20
RENAL	3	**20**
- Renal agenesis	1	6,6
- Ectopic kidney	1	6,6
- Kidney malformation	1	6,6
LIMB	6	**40**
- Radius/ulna anomalies	3	20
- Thumb anomalies	3	20

We also evaluated the presence of non-VACTERL-type malformations. In 9 VACTERL-patients we observed associated non-VACTERL-type anomalies and specifically 1 duodenal atresia, 2 digital anomalies, 1 venous anomalies (persistent left superior vena cava with dilatation of the coronary sinus), 3 single umbilical arteries, 2 genital abnormalities (undescended testis and micropenis), 2 developmental defects of the oropharyngeal region (cleft palate, microretrognathia and high arched palate), 1 respiratory system anomalies, 3 nervous system abnormalities (microphthalmia, coloboma, ventriculomegaly of the occipital horn).

The remaining 10 infants with EA did not have a fully expressed VACTERL phenotype: in 4 newborns the EA was exclusively associated with non-VACTERL-type defects, while in 6 patients EA was associated with only one of the major malformations typically required by the inclusion criteria. In these 6 infants (3 males and 3 females) not fully encountering the inclusion criteria, the most prevalent VACTERL-type defects were the cardiac ones, observed in 3 patients (one ASD and two VSD) and in 2 of them other malformations were not part of the phenotype (duodenal atresia, single umbilical artery, V finger clinodactyly and microcephaly). In the other 3 patients (out of six) we observed separately one with anal anteriorization, one with limb anomalies (supernumerary thumb) and venous abnormalities (portal-vein thrombosis), and one with vertebral anomalies and V finger clinodactyly. Instead in the other 4 babies with EA associated exclusively with non-VACTERL-type defects we observed one with duodenal atresia, another one with venous anomalies (azygos vein ecstasies) and two with congenital hypothyroidism (Additional file 1).

## Discussion

In our study, the cardiovascular system was the most common affected district in association with EA, followed by spine, anal region, limbs and kidney. These results have been compared to those of previously published studies. Solomon et al. reported a higher prevalence of cardiac malformations (80%) followed by vertebral anomalies (78%), renal abnormalities (72%), anal atresia (55%), esophageal atresia (52%) and limb anomalies (47%) [20]. Cardiac anomalies were the most common defects and vertebral anomalies were frequent as well as in our study although, on the other hand, renal malformations were present in 72% of cases, according to other reports, compared to 20% in our population. Indeed, this study included all cases with any three or more of the six main defects, although not necessarily EA/TEF.

We enrolled only those patients with EA/TEF and at least two other typical defects within the spectrum of VACTERL association. There are only a few articles in the literature reporting similar data [15,21,23-25]. de Jong et al. used the same inclusion criteria and reported a higher prevalence of vertebral/rib anomalies (68,9%) and cardiovascular defects (65,6%), followed by anal atresia (42,2%), renal abnormalities (35,6%) and limb anomalies (32,2%) [21]. In this study, both vertebral anomalies and cardiac malformations were the most common VACTERL-type defects, while there are differences about other ones, especially anal atresia and kidney malformations.

The frequency of non-VACTERL-type anomalies varies in the literature: it has been reported a rate of 70% [22] versus 57% reported by other studies [15,22] and much lower percentages by other authors [22]. Anyway, once again it is difficult to compare the results, due to the high heterogeneity of the inclusion criteria chosen for patient selection in different studies. In our study 9 (60%) of 15 newborns with VACTERL association presented non-VACTERL-type anomalies (Table 2).

**Table 2:** **Frequency of non-VACTERL-type abnormalities in 15 patients with VACTERL association and in 10 patients with EA according to de Jong criteria**[18]

**Non-VACTERL-type anomalies**	**de Jong et al. VACTERL patients**	**Palermo NICU VACTERL patients**	**Palermo NICU EA patients**
	**(≥ 3 anomalies)**	**(≥ 3 anomalies)**	**(< 3 anomalies)**
Number of patients	90	15	10
% cases with associated anomalies	70%	60%	70%
Single umbilical artery	20%	20%	20%
Duodenal atresia/stenosis	8,90%	6,6%	20%
Cleft lip and palate	4,40%	13,3%	0
Genital anomalies	7,80%	13,3%	0
Respiratory system anomalies	13,30%	6,6%	10%
Vascular anomalies	7,70%	6,6%	20%
Nervous system anomalies	11,10%	20%	10%
Anomalies of toes	10%	13,3%	20%
Lower limb anomalies	16,7%	6,6%	0%
Hypothyroidism	0%	0%	20%

Ten infants with EA and other multiple malformations did not met the criteria for VACTERL diagnosis. Seven of them (70%) showed non-VACTERL-type anomalies with similar percentages compared with our VACTERL population. The SUA malformation was the most common non-VACTERL-type anomaly found in our population, with a percentage of 20% in VACTERL infants and 20% in infants with EA and other malformations. Other studies reported frequency ranges from 8 to 70% [6,21,23,24], supporting the opportunity to extend the acronym with the letter S (VACTERLS), in spite of including it in the vascular anomalies (V) [2]. Another non-VACTERL-type anomaly which is frequently observed in VACTERL patients is duodenal atresia (8,9% [21], 8% [23,24]), which has been reported in 1 of our VACTERL-patients (6,6%) and 2 (20%) of our infants with EA and other malformations. Two VACTERL newborns (13,3%) presented cleft palate, comparing with 4,4% [21] and 13,2% [15] of other studies. Two newborns (13,3%) showed genital abnormalities (undescended testis and micropenis) against the 7,8% reported in literature [21]. Even the abnormalities of the fingers (ectrodactyly with duplication and/or aplasia of phalanges) are found in 20% of patients with EA associated with other non-VACTERL-type malformations and in the 13,3% of VACTERL infants. We have noticed the presence of venous malformations such as ectasies, dilatation or thrombosis, mainly involving the portal vein, vena cava or azygos vein in 20% of infants with EA and multiple malformations and in 6,6% of VACTERL-patients, percentages similar to those reported in the literature (7,7% [21]). We found abnormalities in the nervous system (such as ventriculomegaly, microcephaly, coloboma and microphthalmia) in 3 neonates (20%) with VACTERL association and 1 (10%) with EA and multiple malformations, according to other reports in the literature [21]. Hypothyroidism was present in 2 infants (20%) with EA and multiple malformations and respiratory system anomalies in 1 newborn (10%) with EA and multiple malformations and in 1 (6,6%) with VACTERL association. Finally, we found lower limb anomalies in 1 neonate (6,6%) with VACTERL association.

**Figure 2:**
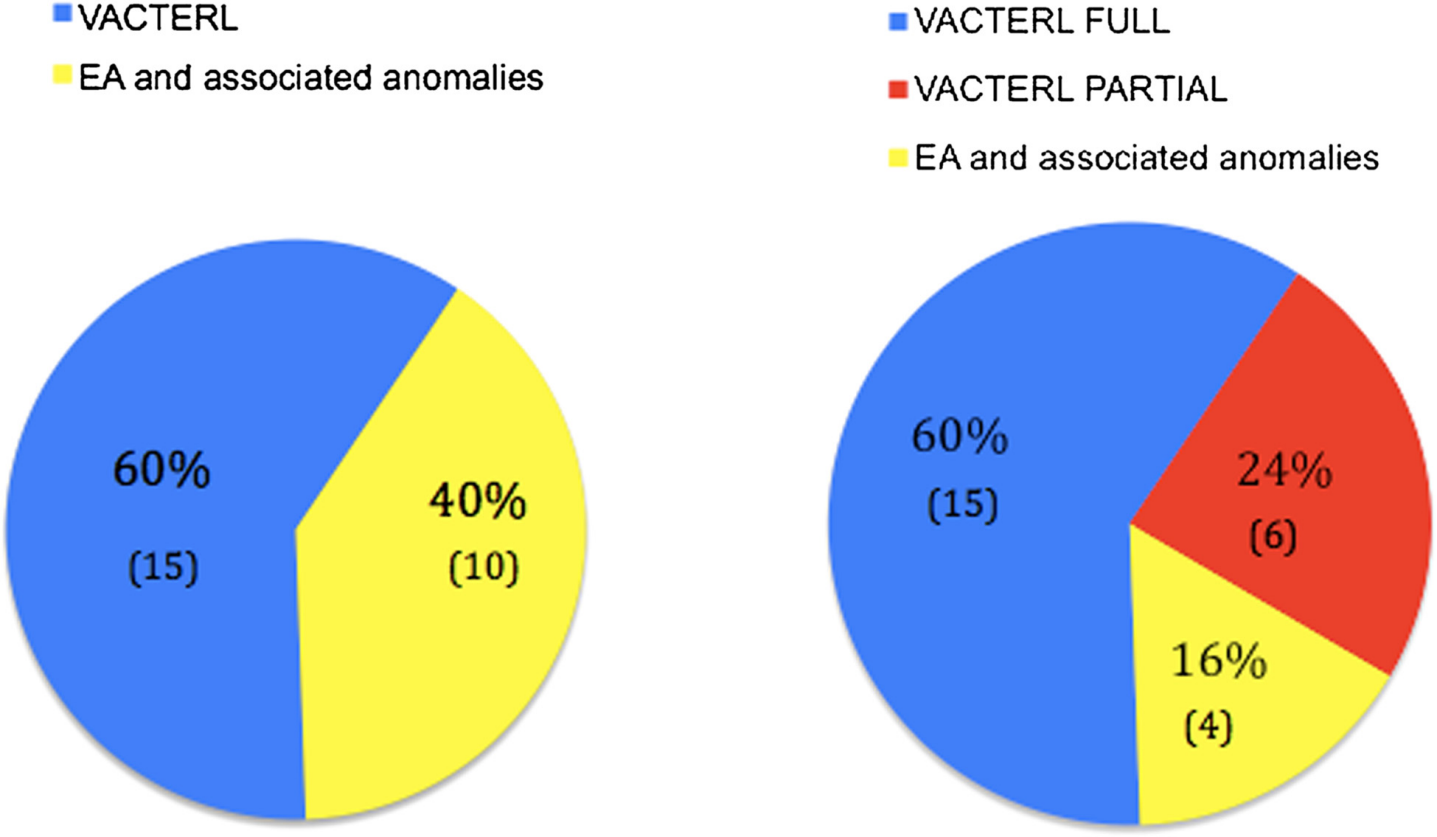
**Partition of patients in three groups.** Identification of three clusters of patients: VACTERL full phenotype, VACTERL partial phenotype and EA with only non-VACTERL-type defects.

**Figure 3:**
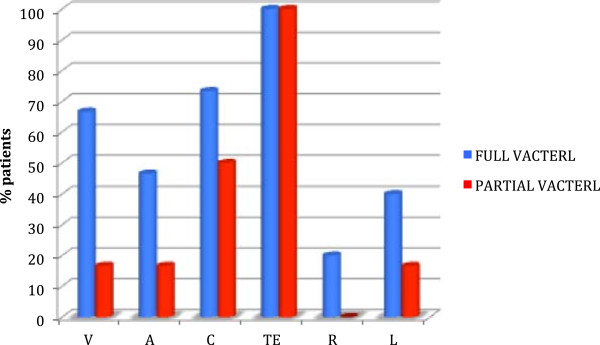
**Comparison between patients with VACTERL full phenotype and VACTERL partial phenotype.** There are several overlapping data for the typical VACTERL malformations. V, vertebral anomalies; A, anal atresia; C, cardiovascular anomalies; TE, tracheoesophageal fistula and/or esophageal atresia; R, renal anomalies; L, limb anomalies.

**Figure 4:**
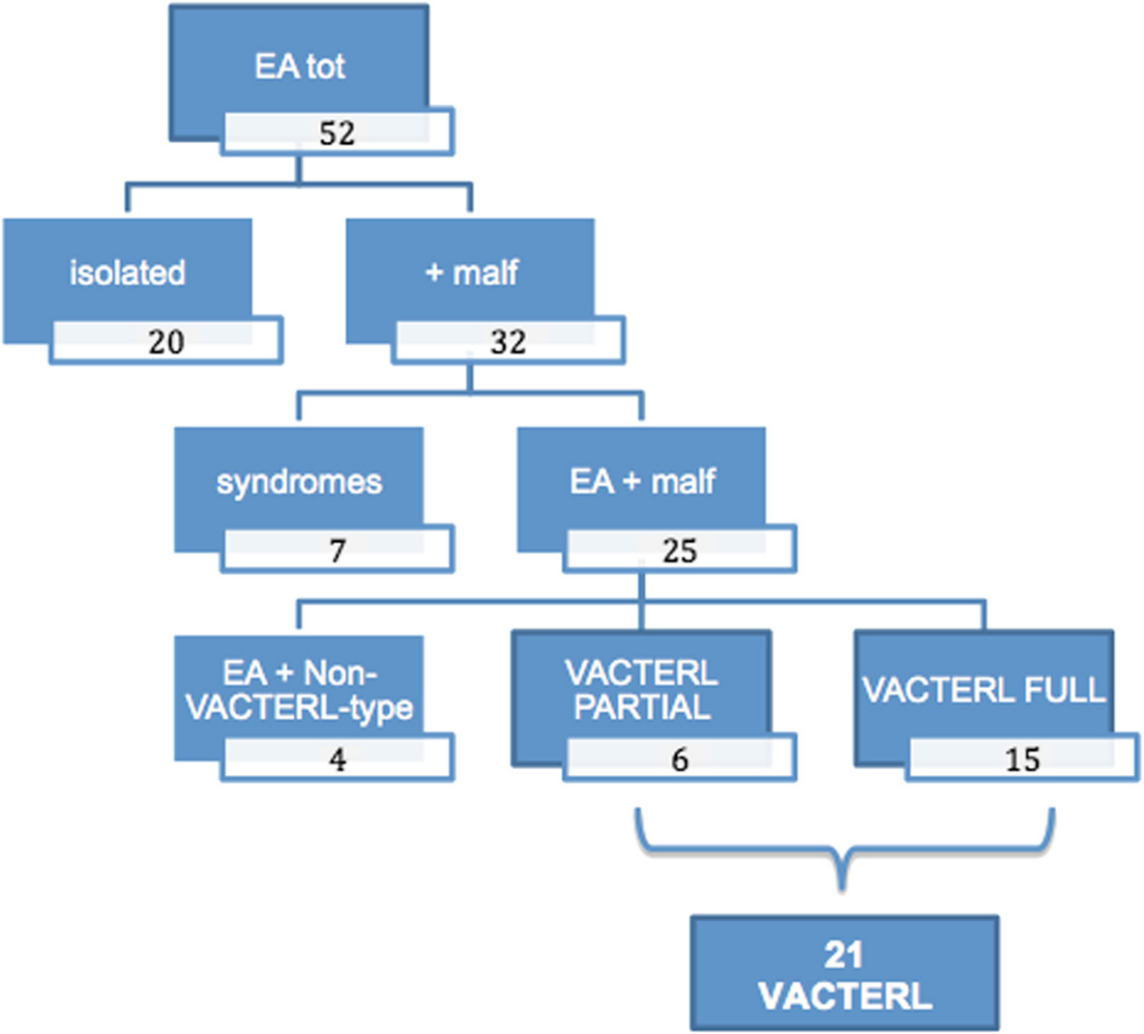
Flow-chart showing patient group composition.

It is interesting to point out that between the two initially disjointed groups, VACTERL-patients with *full phenotype* and infants with EA and multiple malformations, there are several overlapping data, both for the most typical malformations associated with EA and for the non-VACTERL-type anomalies. On these bases, we can actually define three clusters of patients (Figure 2), identifying an additional subgroup with patients who do not show a *full phenotype*, therefore do not meet the classic diagnostic criteria but differ from infants having an EA associated only with non-VACTERL-type defects (Table 3, Figure 3). There 6 patients, presenting only two of the major components of the acronym VACTERL, can be considered at the extreme border of the VACTERL spectrum and can be defined as having a *partial phenotype* (Figure 4).

**Table 3:** VACTERL-type and non-VACTERL-type malformations in three clusters of our cohort

**Anomalies**	**Full phenotype**		**Partial phenotype**		**Non-VACTERL associated anomalies**	
	**(15)**		**(6)**		**(4)**	
V	10	66,6%	1	16,6%	0	0%
A	7	50%	1	16,6%	0	0%
C	10	71,4%	3	50%	0	0%
TE	14	100%	6	100%	4	100%
R	3	21,4%	0	0%	0	0%
L	6	42,8%	1	16,6%	0	0%
SUA	3	21,4%	1	16,6%	1	0%
Duodenal atresia	1	7%	1	16,6%	1	25%
Cleft lip and palate	2	14,2%	0	0%	0	0%
Genital anomalies	2	14,2%	0	0%	0	0%
Hypothyroidism	0	0%	0	0%	2	50%
Vascular anomalies	1	7%	1	16,6%	1	25%
Nervous system anomalies	3	21,4%	1	16,6%	0	0%
Anomalies of toes	2	14,2%	2	33,3%	0	0%
Lower limb anomalies	1	7%	0	0%	0	0%

*V* vertebral anomalies, *A* anal atresia, *C* cardiovascular anomalies, *TE* tracheoesophageal fistula and/or esophageal atresia, *R* renal anomalies, *L* limb anomalies, *SUA* single umbilical artery.

Given that the etiology of VACTERL association is still unknown, the diagnosis is only possible evaluating the phenotypical features, thanks to a good skilled physician. Consequently, these infants with partial phenotype may still belong to the VACTERL association, even though they present a limited clinical spectrum. Analyzing data showed in Additional file 1 it is possible to note the wide spectrum that characterizes patients from the most expressed phenotype to the most restricted one. It is questionable how to split these patients in groups with different prognosis and follow-up schedules. Waiting for more informative molecular-genetic data, it is necessary to avoid restriction of clinical criteria for recruitment, including SUA in the acronym (as already suggested by other authors [8]) and considering other structural abnormalities frequently associated, thus expanding the phenotypic continuum that characterizes the VACTERL association.

It is remarkable to consider that all defects observed in the VACTERL association spectrum, including EA, and most of the non typical associated anomalies take place during the blastogenetic period, the first 4 weeks after conception, in which the embryo is a single developmental field. Most of the associations depends on blastogenetic defects and may show a partial phenotypical overlapping affecting predominantly midline, independently from the etiological basis, due to the common timing and pathogenetic mechanism [11].

## Conclusions

Many associated anomalies may be present in a patients with EA/TEF, which therefore need to be carefully investigated. These complex clinical pictures may represent a recognizable pattern of multiple malformations such as chromosomal or genetic syndromes or associations, mainly VACTERL association. Sometimes a recognizable malformation pattern is not present. The high frequency of non-VACTERL-type anomalies encountered in “*full”* and “*partial”* phenotype patients would suggest the need for an extension of the classical clinical criteria. All these patients, with high phenotypical variability, likely belong to a common wide clinical spectrum and is today very difficult to differentiate clearly distinct groups until more molecular genetic etiologic informations are available. More detailed clinical definition is important to start a proper short and long term follow-up, even in infants with EA/TEF without other VACTERL-type anomalies. The better definition of the extended phenotypical spectrum in VACTERL association allows a more complete diagnostic screening in order to detect even hidden or mild associated malformations, providing a more defined prognosis and follow-up program for the newborn. Future improvements in molecular genetic etiology should allow to assess a genotype-phenotype correlation for such an extended and variable clinical spectrum.

## Competing interests

The authors declare that they have no competing interests.

## Authors’ contributions

All the authors participated in the data collection. SLP and AG designed the study, carried out the data elaboration and coordinated the study. GC and MG revised the manuscript critically for important intellectual content. All authors read and approved the final manuscript.

## Supplementary Material

Additional file 1 Characteristics of 25 patients with esophageal atresia and associated anomalies. Click here for file

## References

[B1] Corsello G Giuffrè M Congenital malformations J Matern Fetal Neonatal Med 2012 25 S1 25 29 10.3109/14767058.2012.664943 22356564

[B2] Solomon BD VACTERL/VATER association Orphanet J Rare Dis 2011 6 56 10.1186/1750-1172-6-56 21846383 PMC3169446

[B3] Quan L Smith DW Bergsma D The VATER association: vertebral defects, anal atresia, tracheoesophageal fistula with esophageal atresia, radial dysplasia The clinical delineation of birth defects. Volume XII. G.I. tract including liver and pancreas 1972 Baltimore: The Williams and Wilkins company 75 78

[B4] Quan L Smith DW The VATER association. Vertebral defects, Anal atresia, T-E fistula with esophageal atresia, Radial and Renal dysplasia: a spectrum of associated defects J Pediatr 1973 82 104 107 10.1016/S0022-3476(73)80024-1 4681850

[B5] Hall BD Suzanne B, Cassidy MD, Allanson JE VATER/VACTERL association Management of genetic syndromes 2010 Third Hoboken, New Jersey: Pubblished by John Wiley & sons Inc. 871 878

[B6] Temtamy SA Miller JD Extending the scope of the VATER association: definition of the VATER syndrome J Pediatr 1974 85 345 349 10.1016/S0022-3476(74)80113-7 4372554

[B7] Nora AH Nora JJ A syndrome of multiple congenital anomalies associated with teratogenic exposure Arch Environ Health 1975 30 17 21 10.1080/00039896.1975.10666626 1109267

[B8] Kaufman RL Birth defects and oral contraceptives Lancet 1973 1 1396 4122792 10.1016/s0140-6736(73)91731-5

[B9] Apold J Dahl E Aarskog D The Vater association: malformations of the male external genitalia Acta Paediatr Scand 1976 65 150 152 10.1111/j.1651-2227.1976.tb16528.x 1258631

[B10] Solomon BD Raam MS Pineda-Alvarez DE Analysis of genitourinary anomalies in patients with VACTERL (Vertebral anomalies, Anal atresia, Cardiac malformations, Tracheo-Esophageal fistula, Renal anomalies, Limb abnormalities) association Congenit Anom (Kyoto) 2011 51 87 91 10.1111/j.1741-4520.2010.00303.x 21235632 PMC3116934

[B11] Corsello G Maresi E Corrao AM Dimita U Lo Cascio M Cammarata M Giuffrè L VATER/VACTERL association: clinical variability and expanding phenotype including laryngeal stenosis Am J Med Genet 1992 1;44 6 813 815 1481853 10.1002/ajmg.1320440619

[B12] Ghirri P Scaramuzzo RT Bertelloni S Pardi D Celandroni A Cocchi G Danieli R De Santis L Di Stefano MC Gerola O Giuffrè M Gragnani GS Magnani C Meossi C Merusi I Sabatino G Tumini S Corsello G Boldrini A Prevalence of hypospadias in Italy according to severity, gestational age and birthweight: an epidemiological study Ital J Pediatr 2009 35 18 10.1186/1824-7288-35-18 19558700 PMC2717564

[B13] Botto LD Khoury MJ Mastroiacovo P Castilla EE Moore CA Skjaerven R Mutchinick OM Borman B Cocchi G Czeizel AE Goujard J Irgens LM Lancaster PA Martínez-Frías ML Merlob P Ruusinen A Stoll C Sumiyoshi Y The spectrum of congenital anomalies of the VATER association: an international study Am J Med Genet 1997 71 8 15 10.1002/(SICI)1096-8628(19970711)71:1<8::AID-AJMG2>3.0.CO;2-V 9215761

[B14] Czeizel A Ludanyi I An aetiological study of the VACTERL-association Eur J Pediatr 1985 144 331 337 10.1007/BF00441773 4076249

[B15] Czeizel A Telegdi L Tusnàdy G Akadémiai Kiadò VACTERL-association Multiple Congenital Anomalies 1988 Budapest: Czeizel, Telegdi, Tusnàdy 247 280

[B16] Kallen K Mastroiacovo P Castilla EE Robert E Kallen B VATER non-random association of congenital malformations: study based on data from four malformation registers Am J Med Genet 2001 101 26 32 10.1002/ajmg.1201 11343333

[B17] Khoury MJ Cordero JF Greenberg F James LM Erickson JD A population study of the VACTERL association: evidence for its etiologic heterogeneity Pediatrics 1983 71 815 820 6835768

[B18] Martinez-Frias ML Frias JL VACTERL as primary, polytopic developmental field defects Am J Med Genet 1999 83 13 16 10.1002/(SICI)1096-8628(19990305)83:1<13::AID-AJMG4>3.0.CO;2-X 10076879

[B19] Rittler M Paz JE Castilla EE VACTERL association, epidemiologic definition and delineation Am J Med Genet 1996 63 529 536 10.1002/(SICI)1096-8628(19960628)63:4<529::AID-AJMG4>3.0.CO;2-J 8826430

[B20] Solomon BD Pineda-Alvarez DE Raam MS Bous SM Keaton AA Vélez JI Cummings DA Analysis of component findings in 79 patients diagnosed with VACTERL association Am J Med Genet A 2010 152A 2236 2244 10.1002/ajmg.a.33572 20683998 PMC2930065

[B21] de Jong EM Felix JF Deurloo JA van Dooren MF Aronson DC Torfs CP Heij HA Tibboel D Non-VACTERL-type anomalies are frequent in patients with Esophageal Atresia/Tracheo-esophageal Fistula and full or partial VACTERL association Birth Defects Res A Clin Mol Teratol 2008 82 92 97 10.1002/bdra.20437 18186125

[B22] Oral A Caner I Yigiter M Kantarci M Olgun H Ceviz N Salman AB Clinical characteristics of neonates with VACTERL association Pediatr Int 2012 54 3 361 364 10.1111/j.1442-200X.2012.03566.x 22300427

[B23] Chittmittrapap S Spitz L Kiely EM Brereton MJ Oesophageal atresia and associated anomalies Arch Dis Child 1989 64 364 368 10.1136/adc.64.3.364 2705799 PMC1791901

[B24] Spitz L Oesophageal atresia Orphanet J Rare Dis 2007 2 24 10.1186/1750-1172-2-24 17498283 PMC1884133

[B25] Keckler SJ St Peter SD Valusek PA Tsao K Snyder CL Holcomb GW 3rd Ostlie DJ VACTERL anomalies in patients with esophageal atresia: an updated delineation of the spectrum and review of the literature Pediatr Surg Int 2007 23 309 313 10.1007/s00383-007-1891-0 17377826

