# Supplementary material for: Esophageal atresia in newborns: a wide spectrum from the isolated forms to a full VACTERL phenotype?
Source: Ital J Pediatr. 2013 Jul 10;39:45. doi: 10.1186/1824-7288-39-45 (PMC3726359; doi:10.1186/1824-7288-39-45)
Supplement: Additional file 1 — Characteristics of 25 patients with esophageal atresia and associated anomalies. [file 1824-7288-39-45-S1.doc]

Additional file 1. Characteristics of 25 patients with esophageal atresia and associated anomalies.

V, vertebral anomalies; A, anal atresia; C, cardiovascular anomalies; TE, tracheoesophageal fistula and/or esophageal atresia; R, renal anomalies; L, limb anomalies; H, hydrocephalus; SUA, single umbilical artery; GA, gestational age in weeks; BW, birth weight in grams; PH, phenotype scale.
